# Acazicolcept (ALPN-101), a dual ICOS/CD28 antagonist, demonstrates efficacy in systemic sclerosis preclinical mouse models

**DOI:** 10.1186/s13075-021-02709-2

**Published:** 2022-01-05

**Authors:** Cindy Orvain, Anne Cauvet, Alexis Prudent, Christophe Guignabert, Raphaël Thuillet, Mina Ottaviani, Ly Tu, Fanny Duhalde, Carole Nicco, Frédéric Batteux, Jérôme Avouac, NingXin Wang, Michelle A. Seaberg, Stacey R. Dillon, Yannick Allanore

**Affiliations:** 1 Université Paris Descartes, Sorbonne Paris Cité, INSERM U1016, Institut Cochin, CNRS UMR8104, Paris, France; 2 INSERM UMR_S 999, Le Plessis-Robinson, France; 3 Université Paris-Sud, Université Paris-Saclay, Le Kremlin-Bicêtre, France; 4 Assistance Publique–Hôpitaux de Paris (AP–HP), Hôpital Universitaire Paris Centre (HUPC), Centre Hospitalier Universitaire (CHU) Cochin, Service d’immunologie biologique (Professeur Batteux), Paris, France; 5 Université Paris Descartes, Sorbonne Paris Cité, Service de Rhumatologie A, Hôpital 27 rue du Faubourg Saint-Jacques, Cochin, 75014 Paris, France; 6 Alpine Immune Sciences, Seattle, WA 98102 USA

**Keywords:** Systemic sclerosis, Dermal fibrosis, Pulmonary fibrosis, Pulmonary hypertension, Costimulation blockade, ICOS, CD28

## Abstract

**Background:**

Uncontrolled immune response with T cell activation has a key role in the pathogenesis of systemic sclerosis (SSc), a disorder that is characterized by generalized fibrosis affecting particularly the lungs and skin. Costimulatory molecules are key players during immune activation, and recent evidence supports a role of CD28 and ICOS in the development of fibrosis. We herein investigated the efficacy of acazicolcept (ALPN-101), a dual ICOS/CD28 antagonist, in two complementary SSc-related mouse models recapitulating skin fibrosis, interstitial lung disease, and pulmonary hypertension.

**Methods:**

Expression of circulating soluble ICOS and skin-expressed ICOS was investigated in SSc patients. Thereafter, acazicolcept was evaluated in the hypochlorous acid (HOCL)-induced dermal fibrosis mouse model and in the Fra-2 transgenic (Tg) mouse model. In each model, mice received 400 μg of acazicolcept or a molar-matched dose of an Fc control protein twice a week for 6 weeks. After 6 weeks, skin and lung were evaluated.

**Results:**

ICOS was significantly increased in the sera from SSc patients and in SSc skin biopsies as compared to samples from healthy controls. Similar body weight changes were observed between Fc control and acazicolcept groups in both HOCL and Fra-2 Tg mice suggesting a good tolerance of acazicolcept treatment. In mice challenged with HOCL, acazicolcept induced a significant decrease in dermal thickness, collagen content, myofibroblast number, and inflammatory infiltrates characterized by B cells, T cells, neutrophils, and macrophages. In the Fra-2 Tg mouse model, acazicolcept treatment reduced lung collagen content, fibrillar collagen, histological fibrosis score, and right ventricular systolic pressure (RVSP). A reduction in frequency of CD4+ and T effector memory cells and an increase in the percentage of CD4+ T naïve cells in spleen and lung of acazicolcept-treated Fra-2 Tg mice was observed as compared to Fc control-treated Fra-2 Tg mice. Moreover, acazicolcept reduced CD69 and PD-1 expression on CD4+ T cells from the spleen and the lung. Target engagement by acazicolcept was demonstrated by blockade of CD28 and ICOS detection by flow cytometry in treated mice.

**Conclusions:**

Our results confirm the importance of costimulatory molecules in inflammatory-driven fibrosis. Our data highlight a key role of ICOS and CD28 in SSc. Using complementary models, we demonstrated that dual ICOS/CD28 blockade by acazicolcept decreased dermal and pulmonary fibrosis and alleviated pulmonary hypertension. These results pave the way for subsequent research on ICOS/CD28-targeted therapies.

**Supplementary Information:**

The online version contains supplementary material available at 10.1186/s13075-021-02709-2.

## Introduction

Systemic sclerosis (SSc) is a rare autoimmune rheumatic disease characterized by vasculopathy and dysregulation of the immune response, and extensive fibrosis of skin and internal organs [1]. This leads to increased morbidity and mortality of SSc patients mainly due to cardiovascular and pulmonary complications [2]. T cells are a major component of SSc pathophysiology as indicated by their early recruitment in SSc skin [3]. Several studies have shown the contribution of Th2, Th17, Th22, Tfh, and CD8+ subsets to inflammation in blood and skin of SSc patients [4]. Early vascular and immune interactions are supported by the recent findings showing that endothelial cells expressing HLA-DR are targeted by cytotoxic CD4+ cells, leading to their apoptosis and likely remodelling in affected SSc tissue [5]. T cell activation, proliferation, and differentiation are based on an appropriate interaction between T cell costimulation molecules and their receptors on antigen-presenting cells (APC). Costimulation blockade in several SSc murine models has shown to mitigate fibrosis, linking T cell activation, and fibrosis/remodelling development [6].

CD28 and inducible T cell costimulator (ICOS) are closely related T cell costimulatory molecules within the immunoglobulin superfamily that bind, respectively, the ligands CD80 and CD86, and ICOS ligand (ICOSL), and play partially overlapping roles in immunity [7]. Signalling through CD28 and ICOS leads to T cell cytoskeletal remodelling, production of cytokines, enhanced survival, and differentiation [8, 9]. CD28 and ICOS also cooperate in lung mucosa to induce differentiation of Th2 effector cells [10]. The concept of interfering with T cell costimulation to treat autoimmune diseases has been clinically validated with abatacept (CTLA-4-Ig), an approved CD28 pathway inhibitor for rheumatoid arthritis (RA), juvenile idiopathic arthritis, and psoriatic arthritis.

The CD28 pathway inhibitor abatacept was evaluated in a phase II trial (ASSET) in early diffuse cutaneous SSc (dcSSc). Although abatacept was well-tolerated in the ASSET trial, patients treated with abatacept did not experience significantly greater improvements of the modified Rodnan Skin Score (mRSS) than those administered placebo, though some improvements in secondary outcome measures were observed in the abatacept arm [11]. These results suggest that CD28 pathway inhibition alone is insufficient to significantly impact skin disease in dcSSc patients.

ICOS is not expressed in naïve T cells but is rapidly upregulated after activation and may represent a key pathogenic pathway unaddressed by CD28 antagonism. ICOS appears particularly important for the function of several activated and/or effector T cell subsets, including differentiated types 1, 2, and 17, as well as follicular helper T cells (TFH) [12]. Indeed, activated T cells often downregulate CD28 and/or become less dependent on CD28 costimulation, and CD28-negative T cells accumulate in various inflammatory diseases, correlating with disease activity and lack of responsiveness to abatacept [13–19]. In contrast, ICOS upregulation correlates with disease activity in several inflammatory diseases [20–24], and in preliminary studies, the anti-ICOSL mAb prezalumab (AMG-557) demonstrated some beneficial activity on the arthritis of systemic lupus erythematosus (SLE) (NCT04058028 [25];), as well as on overall disease activity in Sjögren’s syndrome (SjS) (NCT02334306). However, at present no ICOS pathway antagonists have been approved for therapeutic use.

Preliminary data in SSc patients have demonstrated an increase of soluble ICOS in the sera of patients with diffuse cutaneous SSc [26, 27] and of ICOS+ Tfh-like cells in their skin [28]. Studies in SSc mouse models challenged with bleomycin indicated that ICOS-deficient mice were protected from skin and lung fibrosis [29]. In a GVHD model that shares some similarities with SSc, compelling data have revealed a decrease in dermal inflammation and fibrosis after anti-ICOS antibody administration [28]. Taken together, these data suggest a potential role of ICOS in inflammation-driven lung and skin fibrosis.

We hypothesized that a dual-reactive molecule that blocks both pathways, ICOS together with CD28 may be of interest in immune-related diseases; this general approach has since been shown to abrogate ongoing germinal center reactions during an immune response [30]. The blockade of CD28 and ICOS in an acute GvHD mouse model by the novel dual CD28/ICOS antagonist (acazicolcept/ALPN-101) led to improved survival in acazicolcept-treated mice compared to mice receiving a CD28-CD80/CD86 pathway antagonist (belatacept; CTLA-4-Ig) only [31]. These results suggest that co-targeting ICOS and CD28 is a relevant strategy to suppress autoimmune responses and a Phase II clinical trial (NCT04835441) is ongoing to investigate the efficacy of the drug in SLE. Therefore, we herein evaluated the therapeutic effect of acazicolcept on immune responses and related fibrosis in two complementary mouse models mimicking the severe organ damage observed in SSc patients to determine whether such preclinical study may support future development in human SSc.

## Materials and methods

### Animals

Six-week-old female BALB/c mice were purchased from Janvier Laboratory (Le Genest Saint Isle, France), and experiments were conducted in a conventional facility (C75-14-05). Transgenic female Fra-2 (B6.Cg-Tg(H2-K-Fosl2,EGFP)13Wag) mice were bred in a SPF facility (C75-14-02). All mice were housed in ventilated cages with sterile food and water ad libitum. Animals received humane care in compliance with the guidelines implemented at our institution (INSERM and University Paris Descartes).

### Acazicolcept molecule and pharmacological treatment

Acazicolcept (ALPN-101; ICOSL vIgD-Fc, 80 kDa), provided by Alpine Immune Sciences (AIS) (Seattle, WA), is a dual human ICOS/CD28 inhibitor Fc fusion protein [31]. Acazicolcept (produced at KBI Biopharma, Durham NC) and Fc control protein (produced at AIS) were diluted in PBS and injected intraperitoneally twice a week at molar-matched doses of 400 μg/mouse and 267 μg/mouse, respectively. The mouse dosing regimen was identified from prior mouse pharmacokinetic/pharmacodynamic studies as one that provided adequate exposure and disease-modifying activity in multiple mouse models of autoimmunity and inflammation. However, this dosing regimen would not be used directly to predict human regimens due to species- and disease-related differences in multiple factors including target abundance, binding, and clearance.

### HOCL induction of dermal fibrosis and acazicolcept treatment

Dermal fibrosis was induced in 6-week-old BALB/c mice according to the protocol described by Servettaz et al. [32]. A total of 400 μL hypochlorous acid (HOCl) solution was prepared extemporaneously by adding NaClO (9.6% as active chlorine) to KH_2_PO_4_ solution (100 mM, pH 6.2), usually using a 1:100 ratio. The correct amount of NaClO was adjusted to obtain the desired HOCl concentration, defined by the absorbance of the mixture at 292 nm (optical density between 0.7 and 0.9). In total, 200 μL of HOCl solution was injected intradermally into each shaved flank of the mice using a 27-gauge needle, 5 days a week for 6 weeks. Control mice were injected intradermally with 200 μL of sterilized phosphate buffer saline (PBS) into each shaved flank. A total of 100 μL of acazicolcept or Fc control dosing solutions were injected intraperitoneally twice a week during the 6 weeks of HOCl treatment. Mice were divided into the following groups: PBS (*n* = 6), HOCL + Fc control (*n* = 8), and HOCL + acazicolcept (*n* = 8). Mice were euthanized by cervical dislocation after 6 weeks of treatment (Supplementary Figure 1). This experiment has been carried out once.

### Fra-2 transgenic mice and acazicolcept treatment

Transgenic mice expressing the Fra-2 transgene under the control of ubiquitous major histocompatibility complex class I antigen H-2K^b^ promoter develop microangiopathy, systemic inflammation, lung fibrosis, and pulmonary hypertension [33]. These features follow a similar temporal sequence as observed in human SSc. In the lungs, perivascular inflammatory infiltrates and vascular remodelling appear at the 12th week of age and are followed by fibrosis development at 15th week of age [34]. Fra-2 transgenic mice display severe vascular remodelling of pulmonary arteries leading to their intimal thickening and, in the worst case, to obliteration of vessels [35]. Two groups of Fra-2 transgenic female mice were treated starting at 12 weeks of age with intraperitoneal injections of acazicolcept (*n* = 11) or Fc control (*n* = 8) twice a week, for a total of 6 weeks. Mice were euthanized by exsanguination after right ventricular systolic pressure (RVSP) measurement at 18 weeks of age (Supplementary Figure 2). This experiment has been carried out twice.

### Acazicolcept serum measurement

The concentration of acazicolcept was measured in serum samples collected 24 h after the 8th or 13th dose in the HOCL model, or after the 10th and 13th dose in the Fra-2 Tg model, using an ELISA method developed at Alpine Immune Sciences. Acazicolcept was captured by Fc-specific donkey anti-human IgG antibody (Jackson ImmunoResearch), immobilized onto a 96-well microtiter plate and detected with F (ab’)_2_ fragment, Fc-specific donkey anti-huIgG:HRP (Jackson ImmunoResearch). A calibration curve was generated for each assay plate using SoftMax Pro data acquisition and analysis software (version 7.1, Molecular Devices).

### Clinical follow-up of Fra-2 mice

Fra-2 transgenic mice developed a disease phenotype requiring their clinical follow-up. Monitoring included weighing the mice once a week for the duration of the experiment. All the mice were scored individually using body weight change and observation of their physical appearance and behavior. Mice received a clinical score of 0 to 3, with 0 = normal; 1 = weight loss < 10%, lack of grooming and behavior minor modifications; 2 = weight loss between 10 and 15%, alopecia and skin lesions, reduced mobility, Raynaud’s syndrome; 3 = weight loss > 20%, ruffled fur, hunched posture, lethargy. If mice reached a clinical score of 3 before the end of the experiment, they were euthanized to respect the 3R rule.

### Skin thickness measurement of HOCL-treated mice

Skin thickness (expressed in millimeters) was assessed using a caliper to measure the dermal thickness of the shaved backs of the mice. The measurement was performed once a week until the end of the experiment.

### Collagen measurement

Collagen content was measured in a 3-mm punch from the back skin of HOCL-treated mice or from lung biopsies (right lobes) of Fra-2 mice using Sircol® soluble collagen assay (Biocolor, UK) according to the manufacturer’s instructions. Collagen content was determined from the slope of the standard curve calculated using known collagen concentrations.

### Immunohistochemistry and immunofluorescence

Paraffin-embedded sections of dorsal skin were obtained from (1) PBS/, HOCL/Fc control-, and HOCL/acazicolcept-treated mice, and (2) healthy or lesional skin biopsies obtained from healthy human controls or SSc patients. After antigen retrieval, blocking and tissue permeabilization with PBS + 0.25% Triton X-100, mouse skin sections were incubated with the following primary antibodies diluted in PBS+0.5% BSA overnight at 4 °C: rat anti-CD3 (Abcam, clone RM0027-3B19, dilution 1/50), rabbit anti-CD68 (Abcam, Polyclonal, dilution 1/250), rat anti-CD20 (Abcam, clone GOT214A, dilution 1/100), rat anti-Ly6G (BD Biosciences, Clone 1A8, dilution 1/500), and rabbit anti-alpha-SMA (Abcam, Clone E184, dilution 1/250). Then, slides were incubated with the following secondary antibodies diluted in PBS + 1% BSA for 1 h at RT: goat anti-rabbit (Pierce, dilution 1/200) and goat anti-rat (Invitrogen, dilution 1/500 for Ly6G and CD20; 1/150 for CD3). Visualization was performed with Dako Liquid DAB+Substrate Chromogen System (Agilent Technologies), and slides were counterstained with hematoxylin (Thermo Fisher) and mounted using aqueous mounting medium (Merck Millipore). For human skin sections, the following primary antibodies diluted in PBS+0.5% BSA were incubated overnight at 4 °C: anti-ICOS (Abcam goat polyclonal, dilution 1/130) and anti-CD3 (Abcam Clone CD3-12, dilution 1/250). Then, slides were incubated with the following secondary antibodies diluted in PBS+1% BSA for 1 h at RT: anti-goat AF594 (Thermo Fisher, dilution 1/200) and anti-rat AF488 (Thermo Fisher, dilution 1/200). Slides were mounted using Vectashield® mounting medium with DAPI (Vector Laboratories, UK). Analysis of the immunostaining was performed using the Lamina Multilabel Slide Scanner (Perkin Elmer, USA). Slide staining analysis was performed with CaseViewer software (version 2.4).

### Histopathologic assessment of dermal fibrosis in HOCL-treated mice and fibrosing alveolitis in the Fra-2 Tg model

Fixed 6-mm skin punch biopsies from HOCL-treated mice or left lung from Fra-2 mice were embedded in paraffin. A 4-μm-thick tissue section was stained with Sirius Red. Slides were scanned with the Lamina Multilabel Slide Scanner. For HOCL skin sections, dermal thickness was evaluated by measuring the distance between the epidermal-dermal junction and the dermal-subcutaneous fat junction at five sites on skin sections by two independent blinded examiners with the CaseViewer software (version 2.4). The mean of the 10 values obtained by the two examiners was calculated for each skin section. For Fra-2 Tg lung sections, the severity of fibrosing alveolitis was semi-quantitatively assessed by examining the entire slide, by two examiners blinded to the treatment. The grading criteria were as follows: 0 = normal lung; 1 = minimal fibrous thickening of alveolar or bronchioalveolar walls; 2–3 = moderate thickening of walls without obvious damage to lung architecture; 4–5 = increased fibrosis with definite damage to lung structure and formation of fibrous bands or small fibrous masses; 6–7 = severe distortion of structure and large fibrous areas, and 8 = total fibrous obliteration [36].

### Nonlinear microscopy and second harmony generation (SHG) processing

A 2-photon Leica SP8 DIVE FLIM (Leica Microsystems GmbH, Wetzlar, Germany) was used for lung and skin tissue imaging. Two lasers at 1040 and 880 nm wavelength were used to generate second harmonic (SHG) and two-photon-excited fluorescence (TPEF) signals, collected by a Leica Microsystems HCX IRAPO 25×/0.95 W objective and two external detectors. Microscopy was performed on 16-μm-thick blank blades of sliced lungs or skin. Five samples of each slice were taken. The SHG score was established by comparing the area occupied by the collagen relative to the sample surface. Image processing and analysis (thresholding and SHG scoring) were performed using ImageJ homemade routine as previously described [37].

### Right ventricular systolic pressure (RVSP) and wall thickness measurement in Fra-2 Tg mice

RVSP was assessed in unventilated mice under isoflurane anesthesia (1.5–2.5%, 2L O_2_/min) using a closed chest technique by introducing a catheter (1.4-F catheter; Millar Instruments Inc., Houston, TX) into the jugular vein and directing it to the right ventricle. After RVSP measurement, blood was collected by direct cardiac puncture leading to mouse sacrifice. The heart and lungs were removed and flushed with 5 mL of buffered saline at 37 °C. The left lung was fixed in paraformaldehyde at 4%. For 10 Fra-2 Tg mice (4 Fc control- and 6 acazicolcept-treated), one lobe of the right lung was collected to perform FACS analysis and other lobes were immediately snap-frozen in liquid nitrogen and kept at − 80°C. The percentage of wall thickness [(2 × medial wall thickness/ external diameter) × 100] was determined after alpha-SMA staining on lung slides by two examiners (M.O. and C.G.).

### Spleen and lung cell isolation for flow cytometry staining

Flow cytometry staining was performed on 4 spleen/lung Fc control-treated mice and on 6 spleen/lung acazicolcept-treated mice. Spleens were collected and crushed on a 70-μm cell strainer. Red cells were removed with ACK Lysing Buffer (Thermo Fisher). 1 × 10^6^ cells were collected for flow cytometry staining. One lung lobe was cut in small pieces and incubated in PBS 10% FBS + Collagenase II (1 mg/mL, StemCell Technologies) and DNase I (0.1 mg/mL, StemCell Technologies) for 1 h at 37 °C. After mechanical dissociation with vortexing, cell suspensions were passed through a 70-μm cell strainer. Red blood cells were removed with ACK Lysing Buffer (Thermo Fisher). A Percoll (Sigma) density gradient was generated by resuspending cells in a 40% Percoll solution and adding an 80% Percoll solution below the 40% solution. The cell ring was collected, and the total lung cell suspension was used for flow cytometry staining.

Spleen and lung cells were incubated with Zombie Dye UV (Biolegend) for 15 min at room temperature. Fc receptors were blocked with the TruStain FcX™ (anti-mouse CD16/32) Antibody (Clone 93, Biolegend) for 5 min on ice. Cells were first incubated with an anti-CD62L-APC/Cy7 antibody (Clone MEL-14, Biolegend) for 20 min on ice. Second, the following antibody mix was incubated with cells for 30 min on ice: anti-CD4 BV711 (Clone GK1.5), anti-CD19-AlexaFluor700 (Clone 6D5), anti-CD3-BV510 (Clone 17A2), anti-CD44-BV605 (Clone IM7), anti-CD28-BV421 (Clone 37.51), anti-CD69-BV650 (Clone H1.2F3), anti-PD-1-PE/Dazzle 594 (Clone 29F.1A12), anti-human IgG Fc-PE (clone M1310G05), and anti-ICOS-APC (Clone 7E.17G9) purchased from Biolegend and anti-CD8-BUV737 (Clone 53-6.7) purchased from BD Biosciences. Stained cells were fixed in PBS 2% PFA. Data acquisition was performed on a BD LSR Fortessa Cytometer, and data were analyzed with FlowJo Software (version 10.7.2).

### ICOS measurement in human serums

ICOS protein was quantified by ELISA in the serum of 161 patients with SSc and 35 healthy age- and sex-matched volunteers using the Human ICOS (CD278) ELISA Kit (Thermo Fisher Scientific™) according to the manufacturer’s instructions.

### Statistics

All data analyses were performed using GraphPad Prism 9 Software. Human data were presented as mean with standard deviation (SD) and analyzed with Student’s *t* test. Mouse data were presented as median with ranges and analyzed by Mann-Whitney Test. Correlation data were analyzed with Spearman’s correlation test. A *p* value of less than 0.05 was considered statistically significant.

## Results

### ICOS expression is increased in serum and skin of SSc patients

**Fig. 1:**
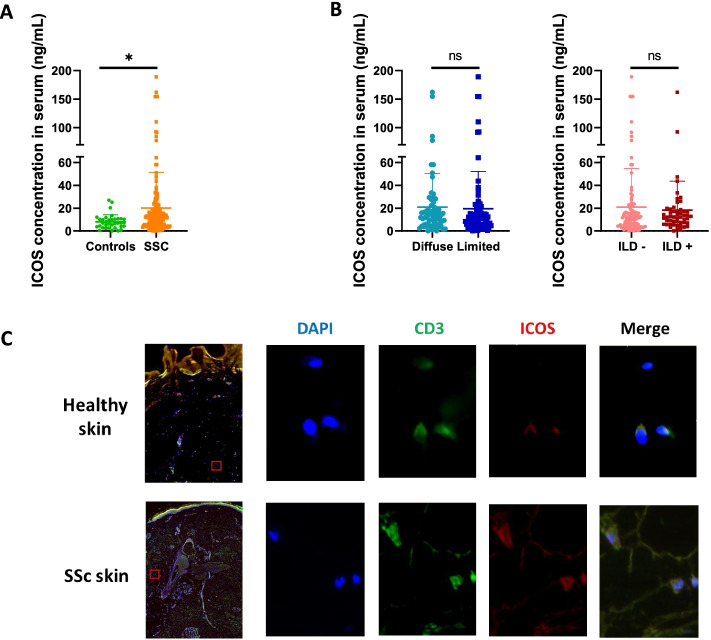
ICOS is increased in SSc serum. **A** ICOS concentration in serum from healthy controls (*n* = 35) and SSc patients (*n* = 161). **B** ICOS concentration in serum of SSc patients divided for diffuse/limited disease or interstitial lung disease or not. **C** Representative images of CD3+ and ICOS+ staining in involved skin from dcSSc of disease duration lower than 3 years and dermal skin from healthy controls (magnification × 10 and × 40). Horizontal lines represent the mean and error bars depict the standard deviation. *p < 0.05 by Student’s *t* test. ns = not significant

We evaluated ICOS expression in the serum of SSc patients (*n* = 161) and healthy controls (*n* = 35). Clinical characteristics of SSc patients are provided in Supplementary Table 1. We observed a higher concentration in SSc patients compared to controls: 20.10 ng/mL ± 31.31 in SSc versus 7.97 ng/mL ± 6.28 in controls (*p* = 0.024) (Fig. 1A). After stratification on skin subsets, we observed no difference between diffuse and limited cutaneous SSc patients: diffuse 20.91 ng/mL ± 29.53 (*n* = 68) versus limited 19.5 ng/mL ± 32.70 (*n* = 93) (Fig. 1B). The sub-group defined by the presence of interstitial lung disease (*n* = 51) was not associated with higher ICOS concentration as compared to patients free of ILD (*n* = 110) (Fig. 1B). No other SSc subset including any other major organ involvement, disease duration, or auto-antibodies was associated with different serum concentrations.

We next investigated CD3+ ICOS+ T cells in healthy controls (*n* = 3) and SSc skin taken from early dcSSc patients (*n* = 3). We observed fewer isolated CD3+ ICOS+ T cells in control skin compared to lesional SSc skin (Fig. 1C).

### Evaluation of acazicolcept efficacy in HOCL-induced dermal fibrosis

#### Acazicolcept prevents HOCL-induced dermal fibrosis

**Fig. 2:**
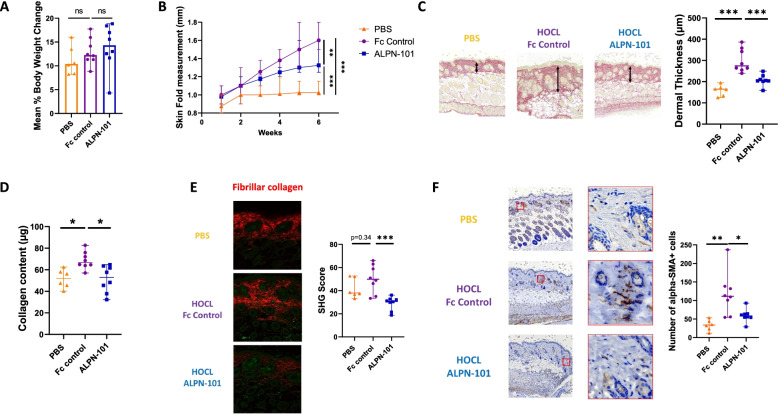
Acazicolcept (ALPN-101) alleviates dermal fibrosis development in the HOCL mouse model. **A** Mean percentage of body weight change calculated between first week and sixth week of treatment. **B** Measurements of skin fold thickness in millimeters from week 1 to week 6, collected weekly. **C** Left: Representative Sirius Red 4 μm-skin sections of PBS-, HOCL/Fc control-, and HOCL/acazicolcept-treated mice (objective × 8). Arrows represents the dermal thickness measurement on each skin section. Right: Mean of five measurements of dermal thickness for each mouse in micrometers. **D** Content of collagen in a 3-mm dorsal skin punch evaluated by Sircol assay in PBS-, HOCL/Fc control-, and HOCL/acazicolcept-treated mice. **E** Left : Representative SHG images of 16 μm dorsal skin sections from PBS-, HOCL/Fc control-, and HOCL/acazicolcept-treated mice. Right : Scoring of fibrillar collagen in PBS-, HOCL/Fc control-, and HOCL/acazicolcept-treated dorsal skin sections. **F** Left: Representative IHC staining of alpha-SMA (brown) in 4-μm skin sections counterstained with hematoxylin of PBS-, HOCL/Fc control-, and HOCL/acazicolcept-treated mice (magnification × 8 and × 100). Right: Number of alpha-SMA positive cells in a 4-μm dorsal skin section from PBS-, HOCL/Fc control-, and HOCL/acazicolcept-treated mice. Mice were divided into three groups: PBS/treated group (*n* = 6), HOCL/Fc control-treated group (*n* = 8), and HOCL/acazicolcept-treated group (*n* = 8). Horizontal lines represent the median with range. *p < 0.05 ; **p < 0.01 ; ***p < 0.001 by Mann-Whitney *U* test

Acazicolcept/HOCL-treated mice had similar body weight changes as observed for the Fc control/HOCL and PBS group (Fig. 2A). As HOCL injections induce skin thickening, we measured dorsal skin folds with a caliper from week 1 to week 6. After 6 weeks of treatment, skin fold thickness was increased by 1.5-fold in HOCL/Fc control-treated mice compared to PBS-treated mice (*p* = 0.0007). Acazicolcept treatment significantly decreased the skin fold thickness by 17.5% compared to HOCL/Fc control-treated mice (*p* = 0.0012) (Fig. 2B).

Skin sections from HOCL/Fc control mice were characterized by marked skin thickening as shown in Fig. 2C. Dermal thickness was 1.7-fold increased in HOCL/Fc control-treated mice compared to PBS-treated mice (*p* = 0.0007). A significant decrease of dermal thickness by 25.5% was observed in HOCL/acazicolcept-treated mice compared to HOCL/Fc control-treated mice (*p* < 0.001) (Fig. 2C).

Skin collagen content was 1.3-fold higher in HOCL/Fc control mice compared to PBS-treated mice (*p* = 0.003). A significant reduction of collagen content by 20.6% was observed in HOCL/acazicolcept-treated mice compared to HOCL/Fc control-treated mice (Fig. 2D).

Fibrillar collagen score as assessed by Second Harmony Generation (SHG) microscopy was 1.3-fold increased in skin sections of HOCL/Fc control-treated mice compared to PBS-treated mice (*p* = 0.34). Acazicolcept treatment decreased fibrillar collagen scores by 38.5% compared to HOCL/Fc control-treated mice (*p* < 0.001) (Fig. 2E).

HOCL/Fc control-treated mice had 3.3-fold higher alpha-SMA myofibroblast counts compared to PBS-treated mice (*p* < 0.001). Acazicolcept treatment decreased the number of alpha-SMA positive cells by 47.7% in dermis compared to HOCL/Fc control-treated mice (*p* = 0.016) (Fig. 2F).

#### Acazicolcept reduces immune cell infiltrate in lesional dermis of HOCL mice

**Fig. 3:**
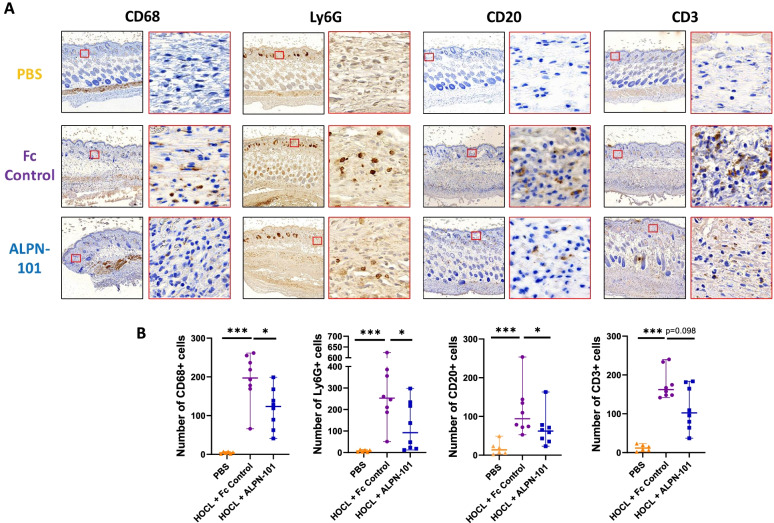
Acazicolcept (ALPN-101) decreased immune cell infiltrates in lesional skin of HOCL-treated mice. **A** Representative IHC staining (brown) of macrophages (CD68+), neutrophils (Ly6G+), B cells (CD20+), and T cells (CD3+) on 4-μm dorsal skin sections counterstained with hematoxylin from PBS-, HOCL/Fc control-, and HOCL/acazicolcept-treated mice (magnification left × 6 right : × 120). **B** Number of CD68, Ly6G, CD20, or CD3-positive cells in a 4-μm dorsal skin section from PBS-, HOCL/Fc control-, and HOCL/acazicolcept-treated mice. Mice were divided into three groups: PBS group (*n* = 6), HOCL/Fc control-treated group (*n* = 8), and HOCL/acazicolcept-treated groups (*n* = 8). Horizontal lines represent the median with range. *p < 0.05; **p < 0.01 ; ***p < 0.001 by Mann-Whitney *U* test. ns = not significant

T cell (CD3+), B cell (CD20+), macrophage (CD68+), and neutrophil (Ly6G+) numbers were 14.2-, 6.9-, 51- and 33.7-fold higher, respectively, in HOCL/Fc control skin compared to PBS skin (*p* < 0.001) pointing to a striking skin immune infiltration in this model (Fig. 3A,B).

Acazicolcept markedly decreased CD68+ macrophages by 40% (*p* = 0.015), Ly6G+ neutrophils by 63.5% (*p* = 0.038), and CD20+ B cells by 34.1% (*p* = 0.049) (Fig. 3B). We also observed a trend for acazicolcept-mediated decreases in CD3+ T cells by 37% in the skin of HOCL/acazicolcept-treated mice compared to HOCL/Fc control-treated mice, although this difference did not reach statistical significance (*p* = 0.098) (Fig. 3B).

### Evaluation of acazicolcept efficacy in the Fra-2 transgenic mouse model

#### Acazicolcept treatment reduces clinical scores in Fra-2 Tg mice

**Fig. 4:**
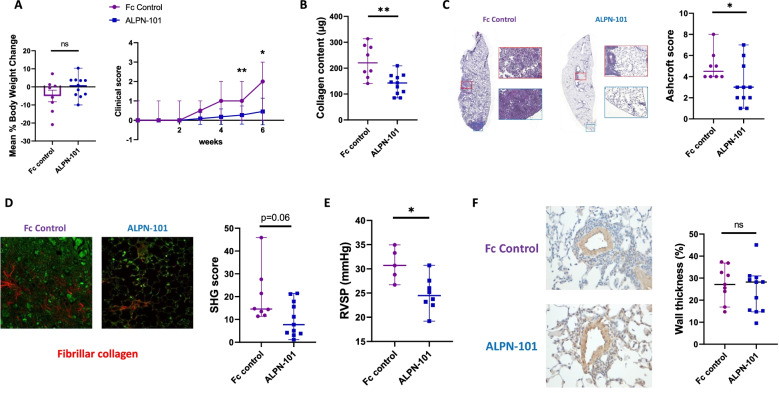
Acazicolcept (ALPN-101) protects against development of lung fibrosis in Fra-2 Tg mice model. **A** Left: Mean percentage of body weight change calculated between first week and sixth week of treatment. Right: Clinical score follow-up during the 6 weeks of treatment based on body weight, coat appearance, and mouse behavior. **B** Content of collagen in a lung fragment (μg) evaluated by Sircol assay in Fc control- and acazicolcept-treated Fra-2 Tg mice. **C** Left: Representative HES 4-μm lung sections of Fc control- and acazicolcept-treated mice (magnification × 8). Right: Ashcroft histological score of Fc control- and acazicolcept-treated Fra-2 Tg mice. **D** Left: Representative SHG images of 16-μm lung sections from Fc control- and acazicolcept-treated Fra-2 Tg mice (magnification × 25). Collagen fibers are colored in red. Right: Scoring of fibrillar collagen in lung sections from Fc control- and acazicolcept-treated mice. **E** Measure of right ventricular systolic pressure (mmHg) of Fc control- and acazicolcept-treated Fra-2 Tg mice after right catheterization of mice. **F** Left: Representative images of vessel remodelling in Fra-2 Tg lungs (magnification × 40) Right: Percentage of wall thickness in Fc control- and acazicolcept-treated Fra-2 Tg mice. Fra-2 Tg mice were divided into two groups: Fc control-treated (*n* = 8) and acazicolcept-treated (*n* = 11). Horizontal lines represent the median with range. *p < 0.05 ; **p < 0.01; by Mann-Whitney *U* test. ns = no significant

In general, the body weight of mice receiving acazicolcept was maintained throughout the experiment compared to mice treated with Fc control that lost body weight with age, though the difference between the groups was not statistically significant (Fig. 4A, left). Clinical scores evaluating weight loss, coat appearance, and mouse behavior decreased by 78.4% (*p* = 0.008) and 72.2% (*p* = 0.012), respectively, at the 5th and 6th week in acazicolcept-treated Fra-2 Tg mice vs. Fc control-treated Fra-2 Tg mice (Fig. 4A, right).

#### Acazicolcept treatment alleviates lung fibrosis and pulmonary hypertension in Fra-2 Tg mice

Acazicolcept treatment decreased collagen content significantly in lungs from Fra-2 Tg mice, by 35.2% (*p* = 0.005) compared to Fc control (Fig. 4B). Lung sections of Fc control-treated Fra-2 Tg mice were characterized by large patchy areas of inflammatory infiltrate and collagen deposition (Fig. 4C). The histological Ashcroft score of fibrosis was significantly reduced by 33.3% (*p* = 0.032) in acazicolcept-treated Fra-2 Tg mice compared to Fc control-treated Fra-2 Tg mice (Fig. 4C). SHG microscopy showed an increase of collagen fibers around lung vessels (Fig. 4D) in Fc control-treated Fra-2 Tg mice. Decreased fibrillar collagen deposition by 47% (*p* = 0.06) was observed in acazicolcept-treated mice compared to Fc control-treated Fra-2 Tg mice (Fig. 4D).

Regarding pulmonary hypertension (PH), a significant reduction (20.3%, *p* = 0.019) of right ventricular systolic pressure (RVSP) was observed in Fra-2 Tg mice treated with acazicolcept compared to Fra-2 Tg mice that received Fc control treatment. However, wall thickness measurement was similar between Fc control-treated Fra-2 Tg mice compared to acazicolcept-treated mice (27% in Fc control versus 28% in acazicolcept).

#### Acazicolcept reduces T cell response in spleen and lungs of Fra-2 Tg mice

**Fig. 5:**
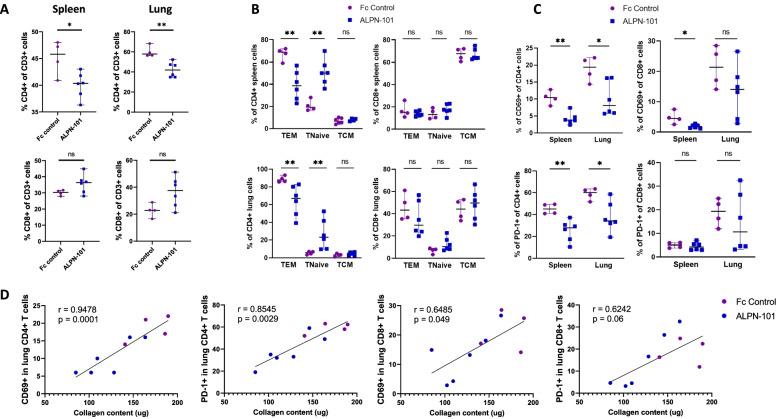
Acazicolcept (ALPN-101) decreased T cell activation in lungs and spleen of Fra-2 Tg mice. **A** Percentages of CD4+ and CD8+ T cells in CD3+ T cells of the spleen and the lung of Fc control- and acazicolcept-treated mice. **B** Percentage of CD4+ or CD8+ effector memory T cells (T_EM_: CD62L− CD44+), naïve T (CD62L+ CD44−), and central memory T cells (T_CM_: CD62L+ CD44+) in spleen and lungs of Fc control- and acazicolcept-treated Fra-2 Tg mice. **C** Percentage of CD69- and PD-1-positive cells within CD4+ or CD8+ subsets in spleen and lungs of Fc control- and acazicolcept-treated Fra-2 Tg mice. **D** Spearman correlation between CD69 and PD-1 expression on lung CD4/CD8 T cells evaluated by flow cytometry and lung collagen content evaluated by Sircol assay. Flow cytometry analysis was performed on 4 spleen/lungs from Fc control-treated mice and on 6 spleen/lungs of acazicolcept-treated Fra-2 Tg mice. Horizontal lines represent the median with range *p < 0.05; **p < 0.01; ***p < 0.001 by Mann-Whitney *U* test. ns = not significant

To evaluate the effects of acazicolcept on T cell responses, we performed flow cytometry analysis by gating on CD4+ and CD8+ populations isolated from the spleen and lungs of treated Fra-2 Tg mice (Supplementary Figure 3). Treatment with acazicolcept significantly reduced the percentage of CD4+ cells by 11.9% in the spleen (*p* = 0.0381) and by 27.6% in the lungs (*p* = 0.009) compared to Fc control-treated Fra-2 Tg mice (Fig. 5A). No significant changes in percentages of CD8+ cells were observed between Fc control- and acazicolcept-treated Fra-2 Tg mice (Fig. 5A). We next investigated the proportions of effector memory T cells (T_EM_), naïve T cells (T Naive), and central memory T cells (T_CM_) based on their differential expression of CD62L and CD44. A significant decrease of CD4+ T_EM_ cells by 43.9 % in spleen and by 23.8% in lungs (*p* = 0.009) in acazicolcept-treated Fra-2 Tg mice was observed compared to Fc control-treated Fra-2 Tg mice. The frequency of CD4+ T naive cells was significantly increased by 2.6 times in spleen and by 4 times in lungs (*p* = 0.009) in acazicolcept-treated Fra-2-Tg mice compared to Fc control-treated Fra-2 Tg mice (Fig. 5B). No differences between Fc control- and acazicolcept-treated Fra-2 Tg mice were observed in the proportions of CD4+ T_CM_, CD8+ TCM, CD8+ T_EM_, or CD8+ naive T cells in spleen and lungs.

Activation of CD4+ and CD8+ T cells was assessed based on the expression of the early activation marker CD69 and the T cell exhaustion marker PD-1. The fraction of CD69-expressing cells was significantly reduced by 63.6% within the CD4+ subset in the spleen (*p* = 0.009) and by 58.2% among CD4+ cells in the lung (*p* = 0.038) upon treatment with acazicolcept compared to Fc control treatment (Fig. 5C). Acazicolcept treatment induced a significant decrease by 60% of CD69-expressing cells among CD8+ spleen cells (*p* = 0.019), but the decrease in CD69-expressing cells within the CD8+ subset in the lung was not statistically significant (*p* = 0.26), compared to Fc control-treated Fra-2 Tg mice (Fig. 5C). Upon treatment with acazicolcept, a significant 38.2% and 43.2% reduction of PD-1-expressing cells was observed within the CD4+ subset in the spleen (*p* = 0.0095) and in the lung (*p* = 0.038) compared to Fc control treatment, respectively. No changes in the frequency of PD-1-expressing cells were detected within the CD8+ subset in the spleen or lung between the two groups of mice.

Interestingly, we detected a strong correlation between the lung collagen content and CD69 or PD-1 expression in lung CD4+ cells (*r* = 0.9478, *p* < 0.001 and *r* = 0.8545, *p* = 0.003, respectively) (Fig. 5D), linking immune activation and extracellular matrix production. Similar findings were observed for CD8+ T cells (Fig. 5D).

#### Acazicolcept serum exposure

**Fig. 6:**
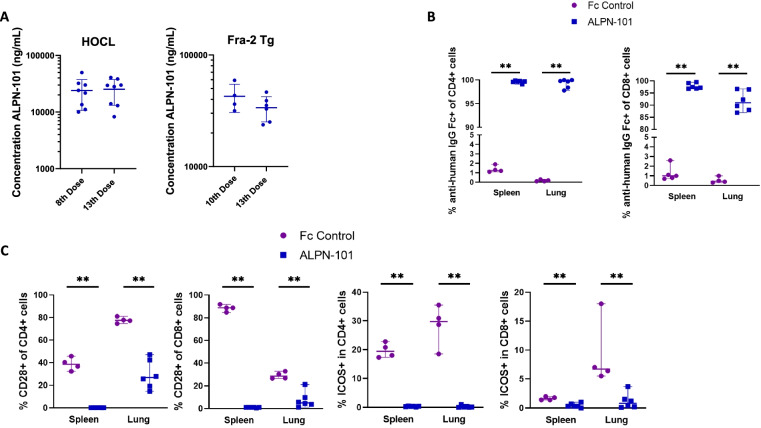
Acazicolcept (ALPN-101) serum exposure. **A** Left : Measurement of acazicolcept concentrations in HOCL-treated mouse serum 24 h after the 8th (*n* = 8) or the 13th dose (*n* = 8). Right: Measurement of acazicolcept concentrations in Fra-2 Tg mouse serum collected 24 hours after the 10th (*n* = 4) or the 13th dose (*n* = 6). Right: **B** Percentage of anti-human IgG Fc-binding cells among CD4+ or CD8+ cells from spleen and lungs of Fc control- (*n* = 4) or acazicolcept (*n* = 6)-treated mice as measured by flow cytometry. **C** Percentage of ICOS- and CD28-positive cells in CD4+ or CD8+ T cells from the lungs and spleen of Fc control- (*n* = 4) and acazicolcept (*n* = 6)-treated mice as measured by flow cytometry. Horizontal lines represent the median with range *p < 0.05; **p < 0.01; by Mann-Whitney *U* test

We observed 24 h after acazicolcept injection similar concentrations between the 10th dose and the 13th dose of acazicolcept in Fra-2 Tg mice (10th dose, mean ± SD 42,630 ± 12,112 ng/mL versus 13th dose 33,728 ± 8591 ng/mL) and between the 8th dose and the 13th dose in HOCL-treated mice (8th dose 24,065 ± 13,359 ng/mL versus 13th dose 25,239 ± 12,090 ng/mL) (Fig. 6A). To track acazicolcept binding to target cells, we stained cells isolated from spleen and lung with anti-human IgG Fc, which can detect the Fc domain of acazicolcept (Supplementary Figure 4). A significant increase of anti-human IgG staining on spleen and lung CD4+ and CD8+ T cells (*p* = 0.009) was observed in acazicolcept-treated Fra-2 mice compared to Fc control-treated Fra-2 mice (Fig. 6B) suggesting acazicolcept was bound to most T cells. Since acazicolcept blocks detection of its targets CD28 and ICOS, we first assessed CD28 expression on splenic and lung T cells by flow cytometry, as a method to track target occupancy (Supplementary Figure 4). We observed a reduced detection of CD28 on spleen CD4+ T cells by 99.7% and spleen CD8 T cells by 98.9 % (*p* = 0.009) in acazicolcept-treated Fra-2 Tg mice compared to Fc control-treated Fra-2 Tg mice. Detection of CD28 was significantly decreased by 66% and by 82.4%, respectively, in lung CD4+ T cells and CD8+ T cells (*p* = 0.0095) isolated from acazicolcept-treated Fra-2 Tg mice (Fig. 6C). We next analyzed ICOS expression on lung and spleen T cells from acazicolcept- and Fc control-treated Fra-2 Tg mice. Detection of ICOS in spleen cells was significantly decreased by 98.2% on CD4+ (*p* = 0.005) and by 81.2% on CD8+ cells (*p* = 0.009) from acazicolcept-treated Fra-2 Tg mice compared to Fc control-treated Fra-2 Tg mice. Like the spleen results, detection of lung ICOS expression was significantly reduced by 99.7% on CD4+ (*p* = 0.009) and by 88.1% on CD8+ (*p* = 0.009) T cells in acazicolcept-treated Fra-2 Tg mice (Fig. 6C). These results demonstrated target engagement of acazicolcept in the spleen and lungs of Fra-2 Tg mice.

## Discussion

We herein showed the overexpression of ICOS in SSc patients and demonstrated the efficacy of acazicolcept, a dual CD28/ICOS antagonist, in two complementary mouse models mimicking the severe features of disease in SSc patients.

The HOCL-induced dermal fibrosis model, based on induction of oxidative stress by hypochlorite, is characterized by dermal inflammation, fibroblast activation, and collagen production [32] as observed in SSc patients [38]. We observed a decrease of dermal thickness, collagen content, myofibroblast number, and inflammatory infiltrate in acazicolcept-treated HOCL-induced mice. However, in this study, we did not observe a decrease of ICOS+ CD3+ T cells in the dermis of HOCL-induced mice following blockade of ICOS with acazicolcept treatment. An investigation of the phosphorylation of molecules involved in ICOS signalling, such as AKT, could be included in future studies. These compelling data support a benefit of acazicolcept treatment in reducing the skin involvement in the HOCL mouse model.

The transgenic Fra-2 mice, in which immune infiltration is followed by pulmonary fibrosis and pulmonary hypertension [33], recapitulates several severe features affecting internal organs of SSc patients [1]. Our study demonstrated that acazicolcept treatment decreased lung fibrosis and collagen content, right ventricular systolic pressure (RVSP), and T cell numbers and activation in Fra-2 Tg mice. It should be noted that while acazicolcept treatment induced a significant decrease of RVSP in these mice, no significant changes were observed in the wall thickness measurement. This may suggest an effect of acazicolcept driven mainly through influence on pulmonary vascular tone by modulating cytokine/chemokine levels [39]. This discrepancy could also be due to the lack of effect of acazicolcept on endothelial cell proliferation and survival. However, one could also speculate that longer exposure or higher doses of acazicolcept treatment might demonstrate an effect on lung vessel remodelling. This hypothesis could be investigated in additional relevant models like the mouse model of chronic hypoxia. It should be emphasized that the magnitude of the effect of acazicolcept is in line with other costimulation blockade therapies already studied in our hands in the Fra-2 Tg model such as abatacept and anti-OX40L antibody [40, 41]. Compared to targeted therapies such as the pan-PPAR agonist IVA337, we observed similar levels of lung fibrosis reduction in acazicolcept-treated Fra-2 Tg mice [42]. Interestingly, our results showed that CD69 and PD-1 expression on CD4+ T cells was positively correlated with lung collagen content, supporting a link between T cell activation and fibrosis development in the Fra-2 Tg model. Our results are aligned with previous studies investigating costimulation blockade in SSc mouse models. Indeed, a decrease of dermal fibrosis and inflammation after ICOS blockade in GvHD-SSc mice [28] or after intradermal bleomycin injections in ICOS^−/−^ mice compared to WT mice [29] was observed. Other costimulation pathways blockade such as CD28-CD80/CD86 and OX40/OX40-L have previously demonstrated decreased pulmonary and dermal fibrosis in SSc mouse models [40, 41, 43].

Recent data have highlighted a role of ICOS in regulatory T cells (Treg). Indeed, it has been demonstrated that ICOS influences Treg accumulation and function in adipose tissue [44]. In parallel, it has been shown that Fra-2 overexpression leads to systemic autoimmunity via the inhibition of Treg development [45]. Therefore, one could speculate that by inhibiting ICOS signalling, Treg recruitment might be enhanced in Fra-2 Tg mouse tissues, potentially contributing to the decrease of inflammation and fibrosis in Fra-2 Tg mice following acazicolcept treatment. However, co-inhibition of the CD28 pathway by acazicolcept complicates comparisons to these studies focused on the role of the ICOS pathway alone. The impact of acazicolcept on human Tregs in vitro and in a humanized mouse model of GVHD has been previously reported [31]. Although acazicolcept and belatacept (CTLA-4-Ig) reduced the proliferation of human Treg and Teff cells in vitro, the relative Treg:Teff ratio was maintained, and consequently the impact of acazicolcept on Treg suppressive activity was negligible. Similarly, acazicolcept reduced Tregs and Teff cells in vivo in the humanized GVHD model, while preserving the Treg:Teff ratio [31]. In future studies, it may be of interest to investigate whether dual ICOS/CD28 blockade can enhance Treg recruitment in the inflamed tissues in acazicolcept-treated Fra-2 Tg mice.

In humans, abatacept (CTLA-4-Ig) has been evaluated in a recent phase II study showing a trend of decreased mRSS in early diffuse cutaneous SSc patients treated with abatacept without reaching significance compared to placebo group [11]. Interestingly, the decline in mRSS was higher in abatacept-treated patients belonging to inflammatory and normal-like skin gene expression subsets compared to the placebo group, providing support for costimulation blockade as a therapeutic strategy for these inflammatory patients.

It is noteworthy that SLE or RA patients treated with CD28/CD80-86 blockade therapies have experienced the emergence of CD28-negative T cell clones that limit the efficiency of these therapies [17]. Acazicolcept, by disrupting both CD28/CD80-86 and ICOS/ICOS-L homodimeric interactions in the T cell synapse, could thus limit emergence of these T cell clones and promote T cell inhibition [31]. It is unlikely that one molecule of acazicolcept can bind CD28 and ICOS simultaneously since CD28-B7 and ICOS-ICOSL interactions are homodimeric and acazicolcept is an Fc dimer; thus, one molecule of acazicolcept likely binds only to CD28 or to ICOS at a time, but not to both. However, it is clear from multiple studies that acazicolcept can functionally inhibit both CD28 and ICOS signalling in parallel to inhibit T cell responses [31, 46].

Our study revealed an increase of soluble ICOS concentrations in a large set of SSc patients, extending data obtained in previous studies [26, 27]. However, we did not observe associations between soluble ICOS levels and mRSS or disease duration, likely due to the lower mRSS scores and higher disease duration in our SSc cohort. It does appear that lesional skin has a higher infiltrate of ICOS+ CD3+ T cells compared to control skin, although this result needs to be confirmed in future studies evaluating a larger number of skin biopsies. One hypothetical concern could be that soluble ICOS in SSc patients might act as a decoy ligand and impair the effectiveness of acazicolcept. However, most reported circulating soluble ICOS levels are in the ng/mL range, while serum concentrations of acazicolcept following treatment are projected to be in the μg/mL range [46]. Moreover, ICOS/ICOS-L interactions typically lead to ICOSL shedding and internalization of ICOS [47] and blocking CD28-CD80/CD86-mediated costimulation has been shown to significantly inhibit ICOS regulation and thus presumably limit soluble ICOS production [48]. Therefore, the action of acazicolcept in patients is unlikely to be impeded by soluble ICOS.

Furthermore, a higher number of circulating T follicular helper cells expressing ICOS was reported in SSc [49] compared to controls; such changes have also been reported in SjS [50], SLE [51], and RA [22]. These human data were complemented by preclinical work reporting reduced disease progression and humoral responses in lupus nephritis and collagen-induced arthritis mouse models after ICOS-L blockade [52]. An anti-ICOSL antibody (AMG557) has been evaluated in patients affected by SjS (NCT02334306) or by active SLE (NCT04058028) but has revealed no statistically significant efficacy in treated patients compared to placebo group, suggesting that inhibition of the ICOS pathway alone may be insufficient to impact disease.

Altogether, the preclinical and clinical results support a role for both the ICOS and CD28 pathways in connective tissue disorders, and our data herein extend the findings to the specific fibrotic phenotype that characterizes SSc.

One limitation from the study herein might be that it does not address whether the dual-specific compound acazicolcept may offer a benefit as compared to single therapies targeting CD28 or ICOS alone; however, each single therapy has already demonstrated its relevance in SSc [28, 29, 40, 43] and in other CTDs [52–59].

The relevance and added value of the dual antagonist approach has been demonstrated in graft-versus-host disease (GVHD) in which acazicolcept demonstrated a benefit in a humanized GVHD mouse model by improving survival and preventing T cell activation and expansion as compared to single costimulatory pathway inhibitors like CTLA-4-Ig [31]. Altogether these and other preclinical data have supported clinical development of acazicolcept (ALPN-101), leading to an ongoing phase 2 trial in SLE (NCT04835441). In order to minimize the number of animals enrolled in our experiments, we decided not to include as controls comparator molecules targeting single pathways, but rather to evaluate the effects of the dual CD28/ICOS antagonist acazicolcept in the specific context of preclinical models of fibrotic CTD. Notably, a recent clinical study revealed for the first time a significant decrease of the dermatological mRSS score in SSc patients treated with a bi-specific antibody targeting IL-4 and IL-13 [60]; these findings further support the strategy of targeting multiple pathways in a complex disease like SSc.

## Conclusion

We have demonstrated evidence of activation of the ICOS pathway both in serum and skin from SSc patients. Furthermore, our study demonstrated that the concomitant blockade of both ICOS and CD28 pathways with acazicolcept leads to a significant decrease in dermal and pulmonary fibrosis in two complementary mouse models of SSc. These data supply a piece to the puzzle of inflammation-driven fibrosis and the role of costimulatory molecules in the setting of SSc. Our results open the door to follow-up studies where clinical data will be required to establish the translation of our findings to patients and to support potential future innovative therapies, especially in the early/inflammatory phase of SSc.

## Supplementary Information

**Additional file 1: Supplementary Table 1**. Clinical characteristics of the SSc cohort. ICOS serum levels were analysed on a cohort of 161 patients affected by SSc.

**Additional file 2: Supplementary Figure 1**. Experimental design of acazicolcept (ALPN-101) treatment in HOCL-induced dermal fibrosis mice. 6-week-old BALB/C female mice were divided into three groups treated with : PBS (n=6), HOCL + Fc control (n=8), or HOCL + acazicolcept (n=8). Dermal fibrosis was induced by subcutaneous HOCL injections five days per week from day 1 to day 42. Test articles (Fc control or acazicolcept) were injected intraperitoneally twice a week. Mice were euthanized 42 days after the first HOCL injection and dorsal skin was collected.

**Additional file 3: Supplementary Figure 2.** Experimental design of acazicolcept (ALPN-101) treatment in Fra-2 Tg mice model. Twelve-week-old female Fra-2 transgenic mice were divided into two groups treated with: Fc control (n=8) and acazicolcept (n=11). Fc control or acazicolcept were injected intraperitoneally twice a week for six weeks. After six weeks of treatment, mice were euthanized, and lungs and spleen were collected for analysis.

**Additional file 4: Supplementary Figure 3.** Gating strategy of T cell sub-populations and activation markers in Fra-2 Tg spleen. Spleen CD3+ T cells were selected in live-gated populations. From total live CD3+ T cells, CD4+ and CD8+ populations were selected. PD-1 (1) and CD69 (2) expression were analysed in CD4+ and CD8+ populations. Among CD4+ or CD8+ T cells, T_CM_ (CD62L+ CD44+, in blue), T_EM_ (CD44+ CD62L-, in purple) and naïve T (CD62L+ CD44-, in green) were selected (3).

**Additional file 5: Supplementary Figure 4.** Gating strategy of ICOS, CD28, and anti-human IgG Fc within CD4+ and CD8+ T cell subsets from Fra-2 Tg spleen. Spleen CD3+ T cells were selected in live-gated populations. From total live CD3+ T cells, CD4+ and CD8+ populations were selected. ICOS (1), anti-human IgG Fc (2) and CD28 (3) staining was analysed in CD4+ and CD8+ populations.

## Data Availability

The datasets used and/or analyzed during the current study are available from the corresponding author on reasonable request. All data generated or analyzed during this study are included in this published article (and its additional information files).

## Abbreviations

AF: AlexaFluor
alpha-SMA: Alpha-smooth Muscular actin
APC: Antigen-presenting cell
BSA: Bovine serum albumin
CD: Cluster differentiation
CPP: Comité de Protection des Personnes
CTD: Connective tissue disease
DAB: Diaminobenzidine
DAPI: 4′,6-diamidino-2-phenylindole
dcSSc: Diffuse systemic sclerosis
ELISA: Enzyme-linked immunosorbent assay
FBS: Fetal bovine serum
Fra-2: Fos-related antigen 2
GvHD: Graft-versus host disease
HES: Hematoxylin eosin saffron
HLA-DR: Human leucocyte antigen-DR
HOCL: Hypochlorous acid
ICOS: Inducible T cell costimulator
ICOSL: Inducible T cell costimulator ligand
IHC: Immunohistochemistry
IL: Interleukin
ILD: Interstitial lung disease
Ly6G: Lymphocyte antigen 6 complex locus GD
mRSS: Modified Rodnan Skin Score
PBS: Phosphate buffer saline
PD-1: Programmed Cell Death 1
PFA: Paraformaldelhyde
PH: Pulmonary hypertension
PPAR: Peroxisome proliferator activated receptor
RA: Rheumatoid arthritis
RT: Room temperature
RVSP: Right ventricular systolic pressure
SD: Standard deviation
SHG: Second harmony generation
SjS: Sjögren’s syndrome
SLE: Systemic lupus erythematosus
SPF: Specific pathogen free
SSc: Systemic sclerosis
TCM: T central memory
TEM: T effector memory
Tfh: T follicular Helper
Tg: Transgenic
Th: T Helper
TPEF: Two-photon excited fluorescence
WT: Wild-type
