# Supplementary material for: Acazicolcept (ALPN-101), a dual ICOS/CD28 antagonist, demonstrates efficacy in systemic sclerosis preclinical mouse models
Source: Arthritis Res Ther. 2022 Jan 5;24:13. doi: 10.1186/s13075-021-02709-2 (PMC8728910; doi:10.1186/s13075-021-02709-2)
Supplement: Supplementary file 1 — Additional file 1: Supplementary Table 1. Clinical characteristics of the SSc cohort. ICOS serum levels were analysed on a cohort of 161 patients affected by SSc. [file 13075_2021_2709_MOESM1_ESM.pptx]

## Slide 1
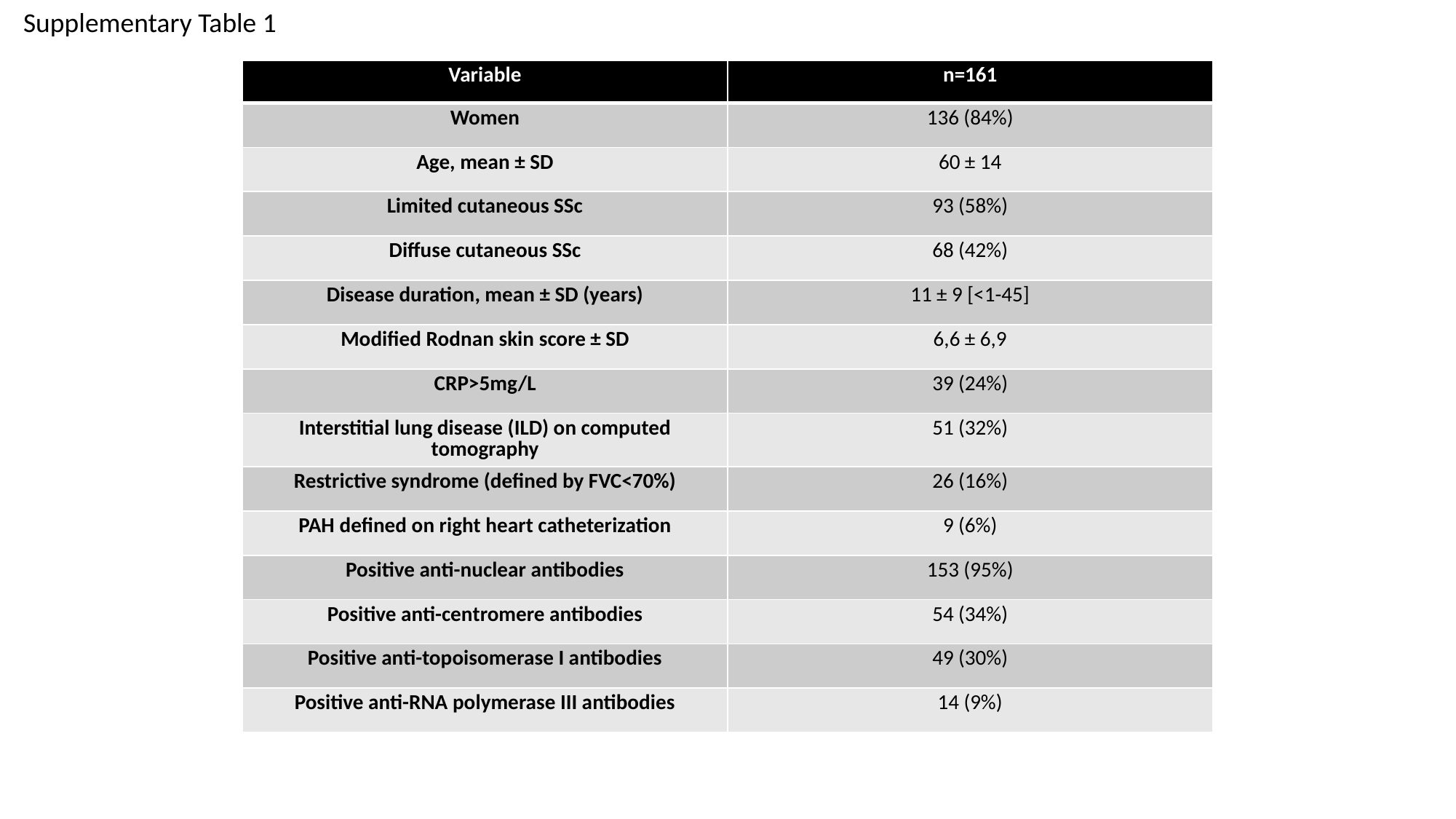

Supplementary Table 1
| Variable | n=161 |
| --- | --- |
| Women | 136 (84%) |
| Age, mean ± SD | 60 ± 14 |
| Limited cutaneous SSc | 93 (58%) |
| Diffuse cutaneous SSc | 68 (42%) |
| Disease duration, mean ± SD (years) | 11 ± 9 [<1-45] |
| Modified Rodnan skin score ± SD | 6,6 ± 6,9 |
| CRP>5mg/L | 39 (24%) |
| Interstitial lung disease (ILD) on computed tomography | 51 (32%) |
| Restrictive syndrome (defined by FVC<70%) | 26 (16%) |
| PAH defined on right heart catheterization | 9 (6%) |
| Positive anti-nuclear antibodies | 153 (95%) |
| Positive anti-centromere antibodies | 54 (34%) |
| Positive anti-topoisomerase I antibodies | 49 (30%) |
| Positive anti-RNA polymerase III antibodies | 14 (9%) |
