# Supplementary material for: Acazicolcept (ALPN-101), a dual ICOS/CD28 antagonist, demonstrates efficacy in systemic sclerosis preclinical mouse models
Source: Arthritis Res Ther. 2022 Jan 5;24:13. doi: 10.1186/s13075-021-02709-2 (PMC8728910; doi:10.1186/s13075-021-02709-2)
Supplement: Supplementary file 2 — Additional file 2: Supplementary Figure 1. Experimental design of acazicolcept (ALPN-101) treatment in HOCL-induced dermal fibrosis mice. 6-week-old BALB/C female mice were divided into three groups treated with : PBS (n=6), HOCL + Fc control (n=8), or HOCL + acazicolcept (n=8). Dermal fibrosis was induced by subcutaneous HOCL injections five days per week from day 1 to day 42. Test articles (Fc control or acazicolcept) were injected intraperitoneally twice a week. Mice were euthanized 42 days after the first HOCL injection and dorsal skin was collected. [file 13075_2021_2709_MOESM2_ESM.pptx]

## Slide 1
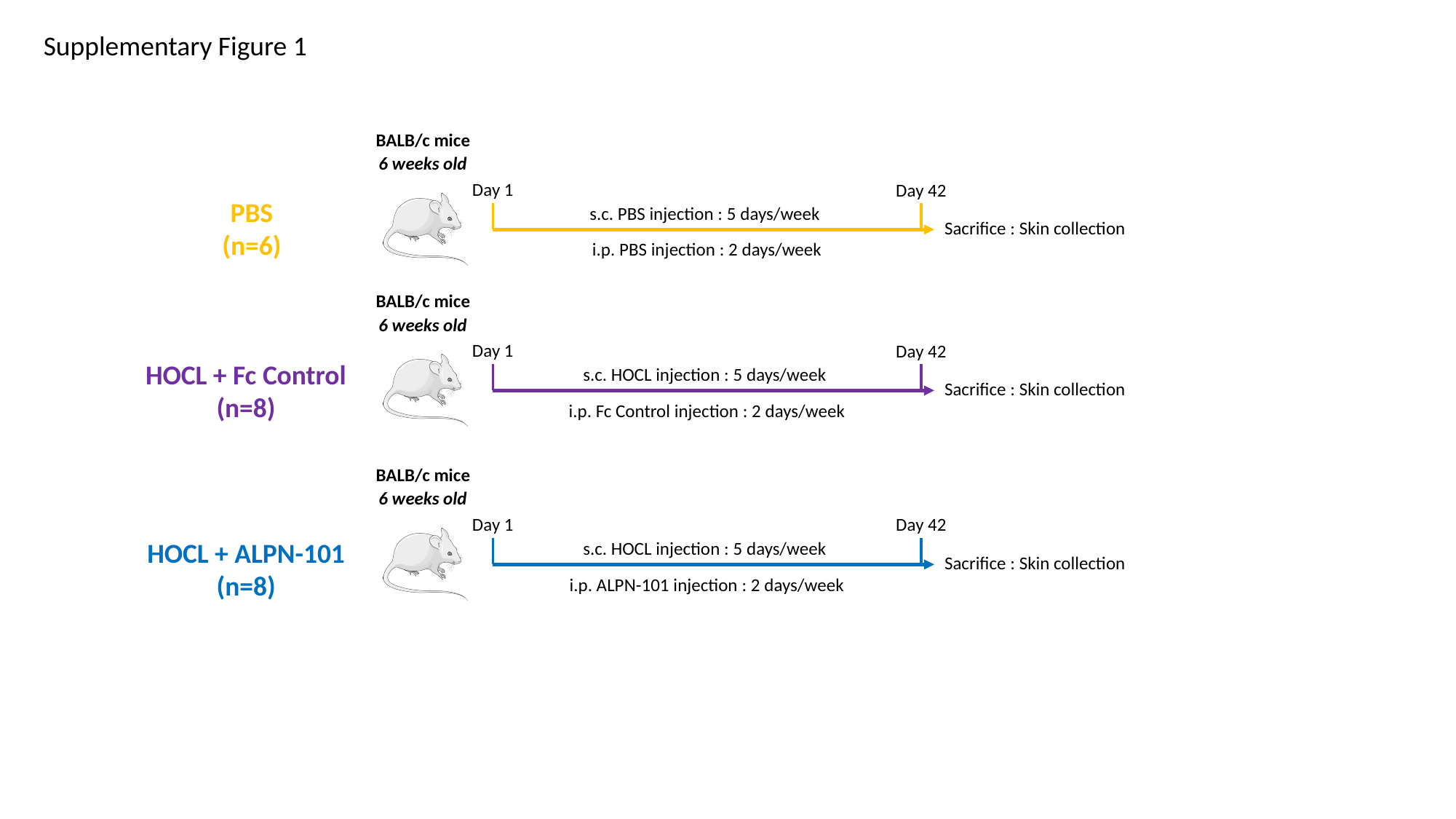

Supplementary Figure 1
BALB/c mice
6 weeks old
Day 1
Day 42
PBS (n=6)
s.c. PBS injection : 5 days/week
Sacrifice : Skin collection
i.p. PBS injection : 2 days/week
BALB/c mice
6 weeks old
Day 1
Day 42
HOCL + Fc Control
(n=8)
s.c. HOCL injection : 5 days/week
Sacrifice : Skin collection
i.p. Fc Control injection : 2 days/week
BALB/c mice
6 weeks old
Day 1
Day 42
HOCL + ALPN-101
(n=8)
s.c. HOCL injection : 5 days/week
Sacrifice : Skin collection
i.p. ALPN-101 injection : 2 days/week
