# Supplementary material for: Acazicolcept (ALPN-101), a dual ICOS/CD28 antagonist, demonstrates efficacy in systemic sclerosis preclinical mouse models
Source: Arthritis Res Ther. 2022 Jan 5;24:13. doi: 10.1186/s13075-021-02709-2 (PMC8728910; doi:10.1186/s13075-021-02709-2)
Supplement: Supplementary file 3 — Additional file 3: Supplementary Figure 2. Experimental design of acazicolcept (ALPN-101) treatment in Fra-2 Tg mice model. Twelve-week-old female Fra-2 transgenic mice were divided into two groups treated with: Fc control (n=8) and acazicolcept (n=11). Fc control or acazicolcept were injected intraperitoneally twice a week for six weeks. After six weeks of treatment, mice were euthanized, and lungs and spleen were collected for analysis. [file 13075_2021_2709_MOESM3_ESM.pptx]

## Slide 1
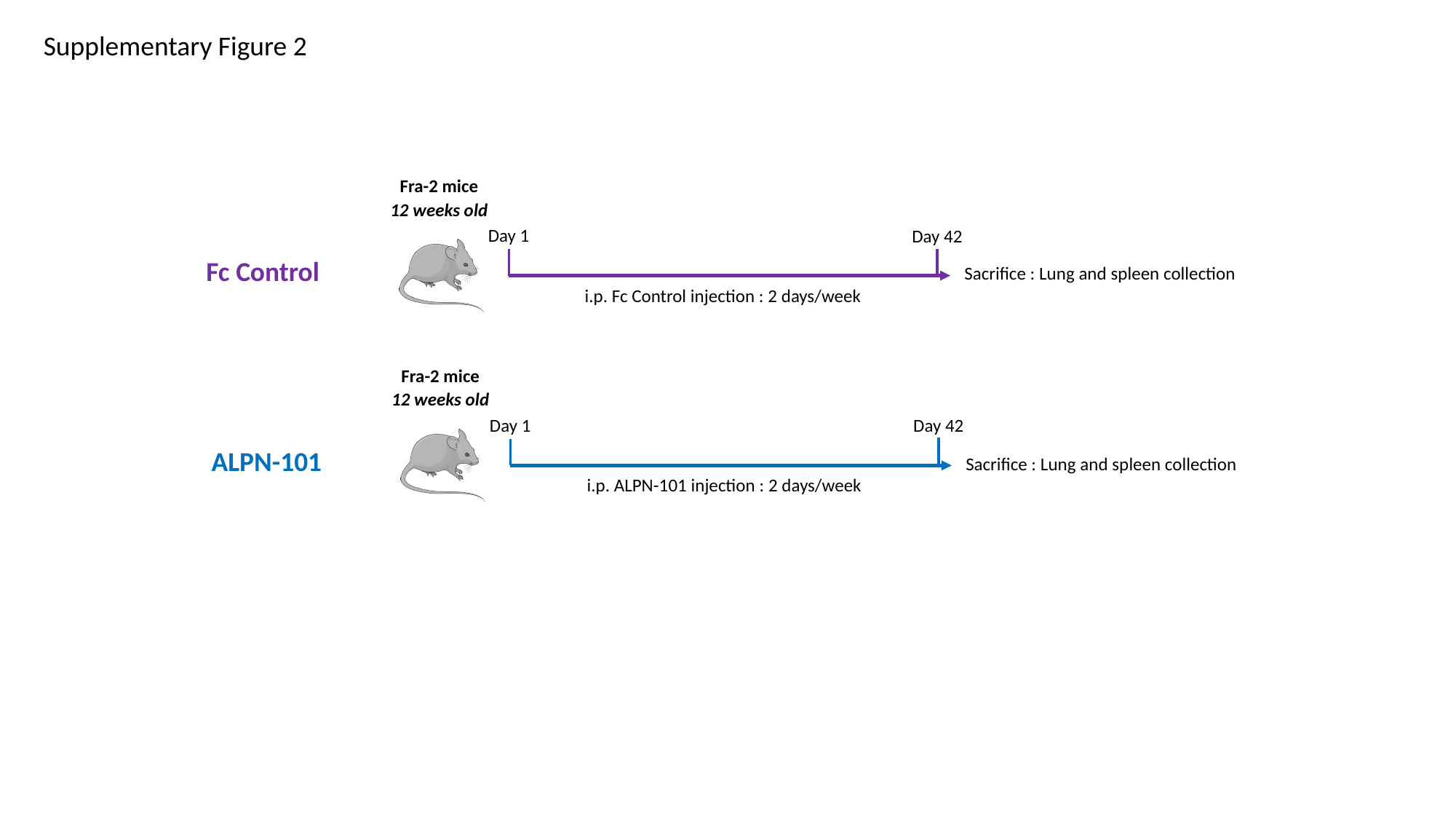

Supplementary Figure 2
Fra-2 mice
12 weeks old
Day 1
Day 42
Fc Control
Sacrifice : Lung and spleen collection
i.p. Fc Control injection : 2 days/week
Fra-2 mice
12 weeks old
Day 42
Day 1
ALPN-101
Sacrifice : Lung and spleen collection
i.p. ALPN-101 injection : 2 days/week
