# Supplementary material for: Acazicolcept (ALPN-101), a dual ICOS/CD28 antagonist, demonstrates efficacy in systemic sclerosis preclinical mouse models
Source: Arthritis Res Ther. 2022 Jan 5;24:13. doi: 10.1186/s13075-021-02709-2 (PMC8728910; doi:10.1186/s13075-021-02709-2)
Supplement: Supplementary file 4 — Additional file 4: Supplementary Figure 3. Gating strategy of T cell sub-populations and activation markers in Fra-2 Tg spleen. Spleen CD3+ T cells were selected in live-gated populations. From total live CD3+ T cells, CD4+ and CD8+ populations were selected. PD-1 (1) and CD69 (2) expression were analysed in CD4+ and CD8+ populations. Among CD4+ or CD8+ T cells, TCM (CD62L+ CD44+, in blue), TEM (CD44+ CD62L-, in purple) and naïve T (CD62L+ CD44-, in green) were selected (3). [file 13075_2021_2709_MOESM4_ESM.pptx]

## Slide 1
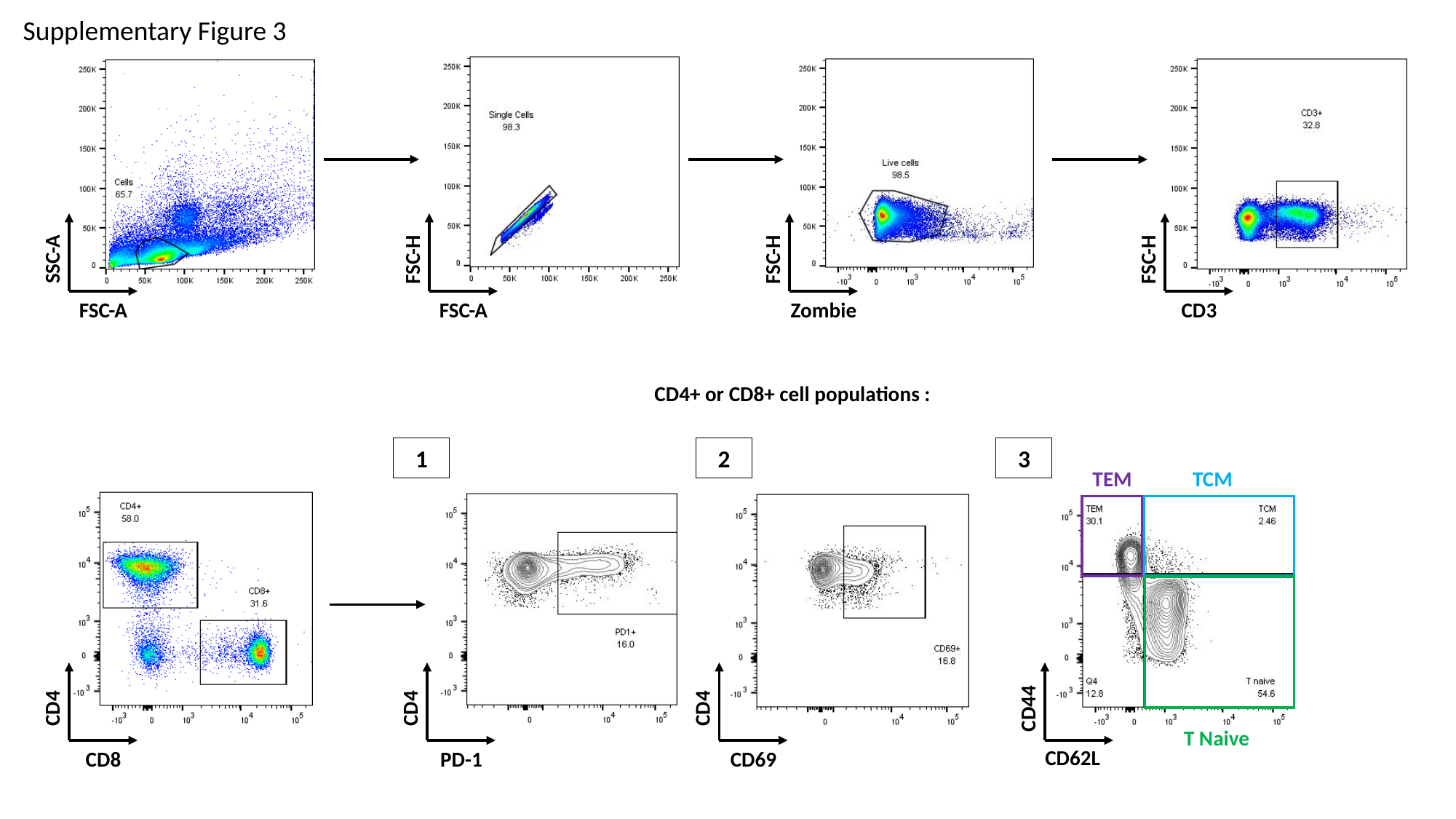

Supplementary Figure 3
SSC-A
FSC-A
FSC-H
FSC-A
FSC-H
Zombie
FSC-H
CD3
CD4+ or CD8+ cell populations :
1
2
3
TCM
TEM
T Naive
CD4
CD8
CD4
PD-1
CD4
CD69
CD44
CD62L
