# Supplementary material for: Acazicolcept (ALPN-101), a dual ICOS/CD28 antagonist, demonstrates efficacy in systemic sclerosis preclinical mouse models
Source: Arthritis Res Ther. 2022 Jan 5;24:13. doi: 10.1186/s13075-021-02709-2 (PMC8728910; doi:10.1186/s13075-021-02709-2)
Supplement: Supplementary file 5 — Additional file 5: Supplementary Figure 4. Gating strategy of ICOS, CD28, and anti-human IgG Fc within CD4+ and CD8+ T cell subsets from Fra-2 Tg spleen. Spleen CD3+ T cells were selected in live-gated populations. From total live CD3+ T cells, CD4+ and CD8+ populations were selected. ICOS (1), anti-human IgG Fc (2) and CD28 (3) staining was analysed in CD4+ and CD8+ populations. [file 13075_2021_2709_MOESM5_ESM.pptx]

## Slide 1
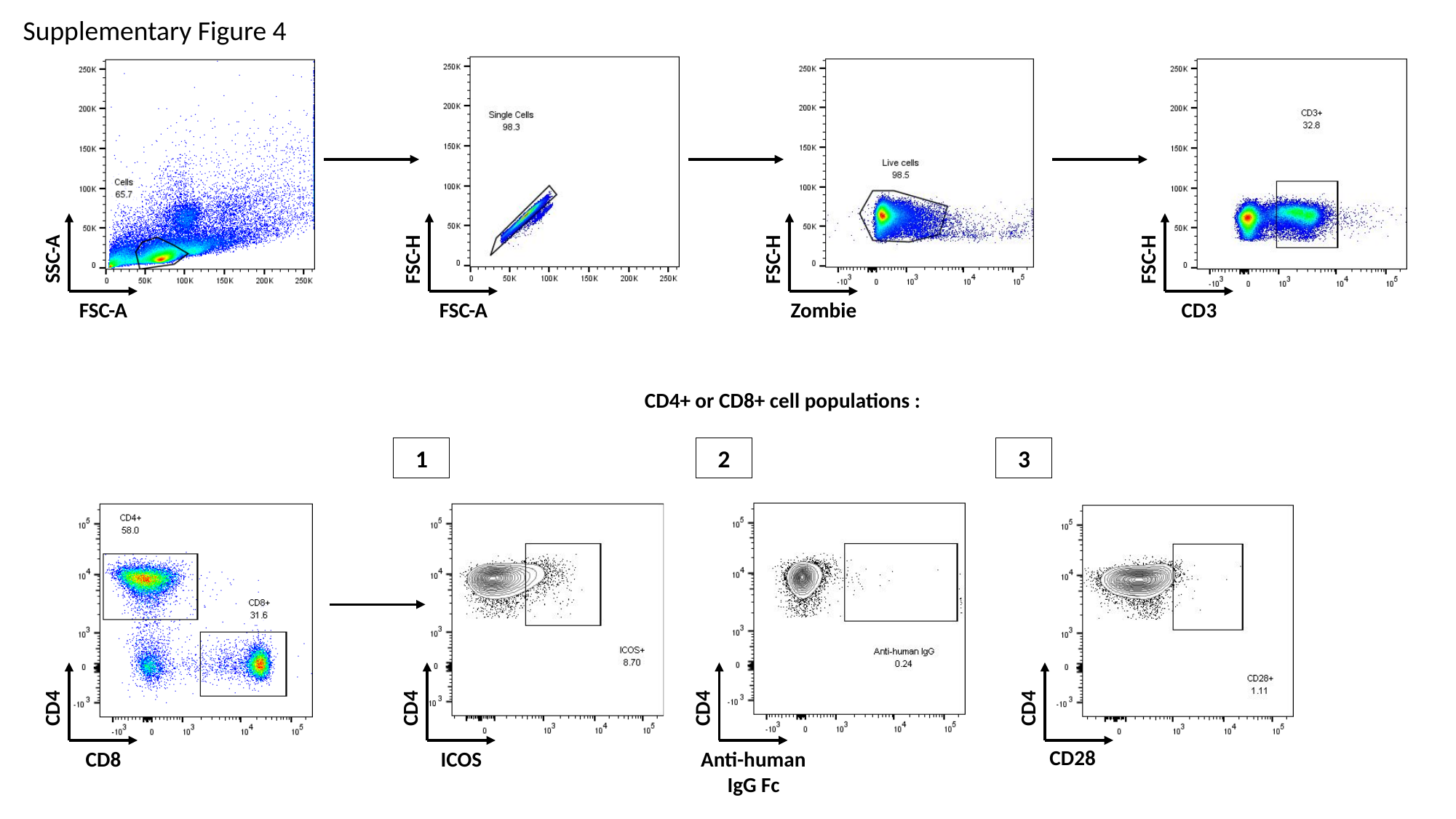

Supplementary Figure 4
SSC-A
FSC-A
FSC-H
FSC-A
FSC-H
Zombie
FSC-H
CD3
1
2
3
CD4
CD8
CD4
ICOS
CD4
Anti-human IgG Fc
CD4
CD28
CD4+ or CD8+ cell populations :
